# Breaking through the mind-body divide: patient priorities for interoception research

**DOI:** 10.1016/j.eclinm.2025.103183

**Published:** 2025-03-31

**Authors:** Lydia J. Hickman, Gabriel Mackie, Beth F. Longley, Hannah S. Savage, Emily Bagley, Hugo Fleming, Rachel Knight, Isabel Lau, Annalise Whines, Sarah N. Garfinkel, Camilla L. Nord

**Affiliations:** a MRC Cognition and Brain Sciences Unit, University of Cambridge, UK; b Insitute of Cognitive Neuroscience, University College London, UK; c Institute of Psychiatry, Psychology and Neuroscience, University College London, UK; d Department of Experimental Psychology, University of Oxford, UK; e Department of Physiology, Anatomy, and Genetics, University of Oxford, UK; f Department of Psychiatry, University of Cambridge, UK

**Keywords:** Psychiatry, Mental health, Interoception, Lived-experience, Patient priorities

## Abstract

**Background:**

Interoception—sensation, interpretation, and prediction of bodily signals—is reliably disrupted across a wide range of mental health conditions. A growing body of evidence suggests that interoception is a putative mechanism, or ‘active ingredient’, of effective psychological and pharmacological treatments. Anecdotally, patients with psychiatric disorders report differences in bodily experiences. However, formal priority setting by people with lived experience of mental health conditions has so far been overlooked in this rapidly expanding research area.

**Methods:**

This article takes a mixed-methods approach to investigate experiences of bodily signals in individuals with mental health conditions and determine patients' research priorities. We recruited two UK samples in the context of an in-person workshop (N = 25) and online (N = 47), between April and July 2024. All contributors had a diagnosis of at least one mental health condition. Using a combination of written contributions and small group discussions, we explored the most relevant bodily sensations for patients’ mental health, how bodily sensations were experienced by patients, and which research priorities were considered most important.

**Findings:**

Patients’ contributions emphasised the multimodal nature of interoception, in particular the importance of less frequently studied modalities such as the stomach and muscle tension, as well as the need to consider the causes and consequences of distressing bodily sensations. We summarise ten key research priorities for patients, spanning three themes: causes, management, and clinical/research approach to interoception in mental health. These priorities include investigating the impact of bodily signals on social contexts, techniques to manage distressing signals, and a shift of approach towards integrating mental and physical health in clinical/research settings.

**Interpretation:**

Together, this broad scoping study establishes new, transdiagnostic, patient-led priorities for the developing field of interoception in psychiatry to ensure future research focusses on the areas of greatest impact for people with mental health conditions.

**Funding:**

This work was supported by a Wellcome Mental Health award to C.L.N. and S.G. (226778/Z/22/Z), intramural funding from the 10.13039/501100000265UK Medical Research Council (MC/UU/00030/12), and a Wellcome Career Development Award to C.L.N. (226490/Z/22/Z). G.M. is funded by an ESRC DTP Studentship (RG84395). This research was also supported by the 10.13039/501100018956NIHR Cambridge Biomedical Research Centre (BRC-1215-20014).

**Research in context**

**Evidence before this study**

Interoception—the sensation, interpretation, and prediction of bodily signals—has gained traction in the field of psychiatry. Experimental evidence indicates that disruptions to interoception are transdiagnostic across mental health conditions, and interoception has been proposed as a putative mechanism, or ‘active ingredient’, of effective psychological and pharmacological treatments. Despite this increased attention, the involvement of people with lived experience of mental health conditions has so far been overlooked. Whilst a number of studies have qualitatively examined specific aspects of interoception in mental health (e.g., its relevance in emotion regulation), a broad scoping study of how people with mental health conditions experience bodily sensations is lacking. In addition, patient-led research priorities for interoceptive research in psychiatry have not been established.

**Added value of this study**

This paper reports mixed-methods lived-experience insight into interoception in mental health from two groups: 25 in-person workshop attendees and 47 online contributors, all of whom had experience of at least one mental health condition. We shed light upon individuals’ experiences of bodily sensations: our findings emphasise the multimodal nature of the connection between the body and mental health, and the relevance of bodily signals that are currently under-researched (e.g., stomach, muscle tension). As well as the processing of bodily signals, contributors also highlighted the need to consider causes and consequences of distressing bodily sensations. We further generated and validated a list of ten research priorities spanning three themes: causes, management, and clinical/research approach to interoception in mental health.

**Implications of all the available evidence**

Our findings combined with existing literature act as strong evidence for the relevance of interoception in mental health conditions. We extend beyond findings from previous experimental studies by providing qualitative evidence of differences in bodily signal processing in people with lived-experience of mental health conditions. To our knowledge, we set out the first generation and validation of patient research priorities for interoception research in psychiatry. We hope that this article provides useful ideas for researchers and clinicians working in this area, including novel insight into how people with mental health conditions experience bodily signals, as well as patient-led priorities for guiding future research.

## Introduction

Interoception refers to the process by which the nervous system senses, interprets, and integrates signals originating from within the body, providing a moment-by-moment mapping of the body's internal landscape across conscious and unconscious levels.^1^ This concept has garnered increased scientific attention in recent years,^2^ not least because differences in interoception appear transdiagnostic across mental health conditions.^1^^,^^3^, ^4^, ^5^, ^6^ The exact differences in interoception vary across conditions including the aspect of interoception affected as well as the bodily signal it refers to. For example, anxiety has been linked to negative evaluation of and increased (negative) attention to bodily signals,^7^ whereas depression has been associated with impairments in interoceptive accuracy.^8^ As a proposed mechanism for such conditions, interoception is a promising target for potential interventions.^9^ However, despite this rapid expansion of interoception research in the mental health sphere, the concept of interoception has only permeated the public consciousness relatively recently.^10^^,^^11^ The involvement of people with lived experience in this field is lacking, both in contributing to knowledge expansion and guiding research priorities. The current article presents a summation of lived experience insights regarding how the body is involved in mental health and, to our knowledge, the first generation and validation of patient research priorities in this area.

Recent collaborations have unified and refined conceptual and measurement approaches in the study of interoception in psychiatry,^1^ but lived experience insights have not yet been incorporated. For example, an ongoing debate is the modalities that should be considered interoceptive. Traditionally, interoception has focused on signals from visceral organs such as the lungs and heart, but more recently some researchers have argued for a broader definition of interoception including sources such as muscle tension and body temperature (see Nord & Garfinkel^9^ for an illustration of these debates). Consequently, much of the research linking interoception to mental health is within the cardiac and respiratory domains,^12^^,^^13^ with a wide array of measures developed for these modalities.^14^^,^^15^ However, it is not clear whether these domains are the most relevant to people with mental health conditions, nor which modalities patients would spontaneously identify when asked to report problems with bodily sensations.

There has been an increased focus in recent years on involving patients and other stakeholders in health research, commonly termed Patient and Public Involvement (PPI), aiming to improve research with their practical experiential expertise. Conducting PPI-led research has many benefits, including ensuring that research is answering questions that are relevant to patients, and designing studies that are more appropriate and accessible for the target population.^16^ The James Lind Alliance, a non-profit organisation aimed at bringing patients, carers and clinicians together, has produced research priorities via their Priority Setting Partnerships for over one hundred specific conditions or situations, from depression to sepsis.^17^^,^^18^ The neurodiversity literature reports lived-experience insights into language preferences^19^ and research priorities (both general^20^ and applied specifically to a context such as disordered eating^21^), something which the mental health literature may draw inspiration from.

Recently, a small number of studies have begun to report qualitative insights into interoception^22^, ^23^, ^24^, ^25^: for example, Zamariola and colleagues^23^ indicate a positive relationship between interoceptive ability and emotion regulation, whilst Neukirch and colleagues^24^ used post-intervention interviews to reveal positive effects on interoceptive awareness following a yoga intervention. PPI work has also begun looking at the impact of bodily signals on mental health in the sphere of circadian rhythm.^26^ However, a broad scoping study of how people with various mental health conditions experience bodily sensations is lacking. As yet, patient-led priorities for the rapidly-expanding field of interoceptive research in psychiatry have not been established. Here, we set out to reduce the mismatch between researcher and patient priorities^27^ by reporting transdiagnostic interoception-related research priorities from people with lived experience of mental health conditions.

## Methods

### Contributors

We recruited two samples consecutively: the first for an in-person workshop (hosted 10th May 2024), and the second for an online data collection procedure (data collected between 28th June and 20th July 2024); recruitment for both samples began on 10th April 2024. These dual strategies enabled us to recruit a wide range of individuals with mental health conditions, including those unable to attend an in-person session. Contributors were recruited via a national PPI panel,^28^ a PPI panel based at Cambridge University Hospitals, and the MRC Cognition and Brain Sciences Unit panel. To recruit a diverse transdiagnostic sample, participation was open to anyone over 18 years old who had ever been diagnosed with any mental health condition. There were no exclusions based on number of mental health diagnoses, physical health conditions, neurodivergence, or other particular background or experiences, including previous PPI experience.

In total, 25 individuals attended the workshop and a further 47 contributed online. With one workshop contributor unable to attend the first half of the session, we had the following sample sizes per analysis: qualitative analyses of experiences of bodily sensations (N = 24), quantitative analyses of experiences of bodily sensations (N = 71), research priority generation (N = 25), research priority validation (N = 47). The sample size for the in-person contributors was constrained by the number of potential facilitators available (N = 5) and our ambition that each group discussion was small enough to consider points in-depth. Given these constraints, we recruited five contributors per facilitated group to enable a thorough discussion, and the subsequent in-depth qualitative analysis of data. The sample size for the online group was determined by the number of interested contributors over a three month period who were unable to attend the in-person workshop (either due to physical barriers to participation or a lack of available workshop places). Table 1 further details for the two samples including demographics and mental health diagnoses. Given high co-occurrence between mental health, physical health and neurodevelopmental conditions,^29^^,^^30^ we did not make exclusions on this basis. As such, these groups were well represented in our sample, with 28% diagnosed with one or more neurodevelopmental condition and 58% diagnosed with at least one physical health diagnosis. Further information is provided in Supplementary Material 1 (Tables S1 and S2). Our procedure was approved by the local Research Ethics Committee (HBREC.2023.01) and contributors were paid £25 an hour, in line with NIHR-recommended rates.^31^ Informed consent was obtained from contributors (either in-person or online for workshop and online samples respectively) and contributors were additionally able to withdraw their data from the study at the end of their participation.

**Table 1:** Demographics and mental health diagnoses of the combined, workshop, and online samples.

Number (N)	Combined		Workshop		Online	
	N = 72		N = 25		N = 47	
Age (mean [SD] range)	44 [17]	19–85	43 [19]	19–85	44 [16]	19–71
Gender						
Woman	52	(72%)	15	(60%)	37	(79%)
Man	17	(24%)	9	(36%)	8	(17%)
Non-binary	3	(4%)	1	(4%)	2	(4%)
Ethnicity						
White	59	(82%)	20	(80%)	39	(83%)
Asian	7	(10%)	2	(8%)	5	(11%)
Black	3	(4%)	2	(8%)	1	(2%)
Multiple	2	(3%)	1	(4%)	1	(2%)
Latino	1	(1%)	0	(0%)	1	(2%)
Diagnosis						
Depression	57	(79%)	18	(72%)	39	(83%)
GAD	31	(43%)	11	(44%)	20	(43%)
PTSD	13	(18%)	2	(8%)	11	(23%)
Other anxiety disorder	11	(15%)	2	(8%)	9	(19%)
Personality disorder	9	(13%)	3	(12%)	6	(13%)
Eating disorder	8	(11%)	2	(8%)	6	(13%)
Panic disorder	8	(11%)	3	(12%)	5	(11%)
OCD	7	(10%)	1	(4%)	6	(13%)
Schizophrenia/Psychosis	7	(10%)	2	(8%)	5	(11%)
Bipolar	6	(8%)	2	(8%)	4	(9%)
Specific phobia	3	(4%)	2	(8%)	1	(2%)
Other	9	(13%)	4	(16%)	5	(11%)
Number of diagnoses						
1	18	(25%)	9	(36%)	9	(19%)
2	29	(40%)	8	(32%)	21	(45%)
3	13	(18%)	6	(24%)	7	(15%)
4	6	(8%)	1	(4%)	5	(11%)
5	4	(6%)	1	(4%)	3	(6%)
6	2	(3%)	0	(0%)	2	(4%)

*Note.* For each demographic category, the table represents count data followed by the percentage in brackets. For diagnosis, count indicates the number of diagnoses of the condition in the sample; number in brackets represents the percentage of contributors in the sample with the condition. Diagnoses in the table represent self-reported diagnoses. For number of diagnoses, count indicates how many contributors in the sample have the specified number of diagnoses; number in brackets is the count as a percentage. The ‘Other’ category contains the following conditions: Adjustment Disorder, Complex PTSD, Cyclothymia, Depersonalisation and Derealisation Disorder, Dissociative seizures, Globus pharyngis, Persistent Delusional Disorder, and Seasonal Affective Disorder.

GAD = Generalised Anxiety Disorder; OCD = Obsessive Compulsive Disorder; PTSD = Post-traumatic Stress Disorder; SD = standard deviation.

### Study design

The study consisted of an in-person workshop with an additional online consultation. The full list of questions asked in each format are available in Supplementary Material 2 (Table S3). The questions were amended and improved following a piloting process with three laypersons who had diagnoses of mental health conditions.

The in-person workshop (N = 25) lasted approximately two hours. It began with soliciting individual free-text responses on how bodily sensations related to patients' mental health (*initial thoughts*: note this preceded any researcher input into the topic). All text responses were collected via polling software Slido (www.slido.com). This was followed by a researcher-led presentation introducing the concept of interoception, in which patients were able to ask questions and clarifications regarding the concept. Whilst this provided contributors with a better understanding of which bodily signals and aspects of interoception may be included in interoception research, findings for specific mental health conditions were not stated to avoid influencing contributors' personal reflections (though note that approaches for improving perception of bodily signals, such as mindfulness and breathing techniques, were mentioned). Individual text responses were then collected again, with contributors selecting and ranking the relevance of various bodily sources to their experience (*experiences of bodily sensations—quantitative*). Next, we subdivided contributors into multiple small-group discussions (consisting of five or fewer contributors, one facilitator, and one note-taker) focussing on contributors’ personal experiences of bodily sensations in the context of mental health (*experiences of bodily sensations—qualitative*). We then collected further text responses regarding mental and physical healthcare (*interactions with the healthcare system*).

The key outcome of our workshop was the generation of patients’ top research priorities. To this end, we asked workshop contributors to indicate what research questions they would most want answered on the topic of bodily sensations/interoception and mental health. These were grouped thematically by two researchers (see below) to produce a list of research priorities (*research priority generation*).

The online consultation (N = 72) was conducted via Qualtrics. It included the same presentation as the workshop (pre-recorded: https://www.youtube.com/watch?v=QRjFl4GSlZU). Online contributors were also asked identical questions on sources of distress and interactions with the healthcare system. Finally, to validate research priorities generated in the workshop, the online sample were asked to rate each topic from zero to ten (where zero = “not at all important” and ten = “extremely important”; *research priority validation*).

### Analyses

#### Qualitative analyses

For qualitative analyses, data for *initial thoughts*, *interactions with the healthcare system*, and *research priority generation* were derived from text written directly by contributors. For the *experiences of bodily* *sensations* section, data took the form of detailed notes written by note-takers during small group discussions. The final set of research priorities (*ten patient-led priorities for interoception research in psychiatry*) were synthesised from the list of research priorities previously generated and rated by contributors.

For each dataset, we conducted a thematic analysis following established protocols.^32^^,^^33^ Two researchers (LJH and GM) independently coded each response with labels and themes. Following this, the two researchers met to resolve any differences, and reviewed the thematic structure of the coding to ensure the nuances of the data were represented. During this stage, we extracted example quotes providing a rich summation of the themes. Finally, we reviewed the contents of each theme to ensure internal consistency and external distinctiveness. This led to the refinement and naming of themes based on their contents, as well as the identification of any relevant sub-themes.

#### Quantitative analyses

For quantitative analyses of *experiences of* *bodily* *sensations* and *interactions with the healthcare system*, we combined data from the workshop and online samples. We calculated summary statistics for the combined samples for ranked and multiple-choice data. For research priority validation, we calculated two summary statistics: (1) the mean rating for each research priority, and (2) the proportion of contributors rating each research priority as below four, four to six, and over six. Missing data points were excluded from analysis: 1.4% of responses to discussing bodily signals in mental health appointments, and 2.4% of research priority ratings. Additionally, 3.7% of sources selected as important were not present in contributors’ ranked data.

### Role of the funding source

Funding sources played no role in the study design; in the collection, analysis, and interpretation of data; in the writing of the report; or in the decision to submit the paper for publication.

## Results

### Initial thoughts

**Fig. 1:**
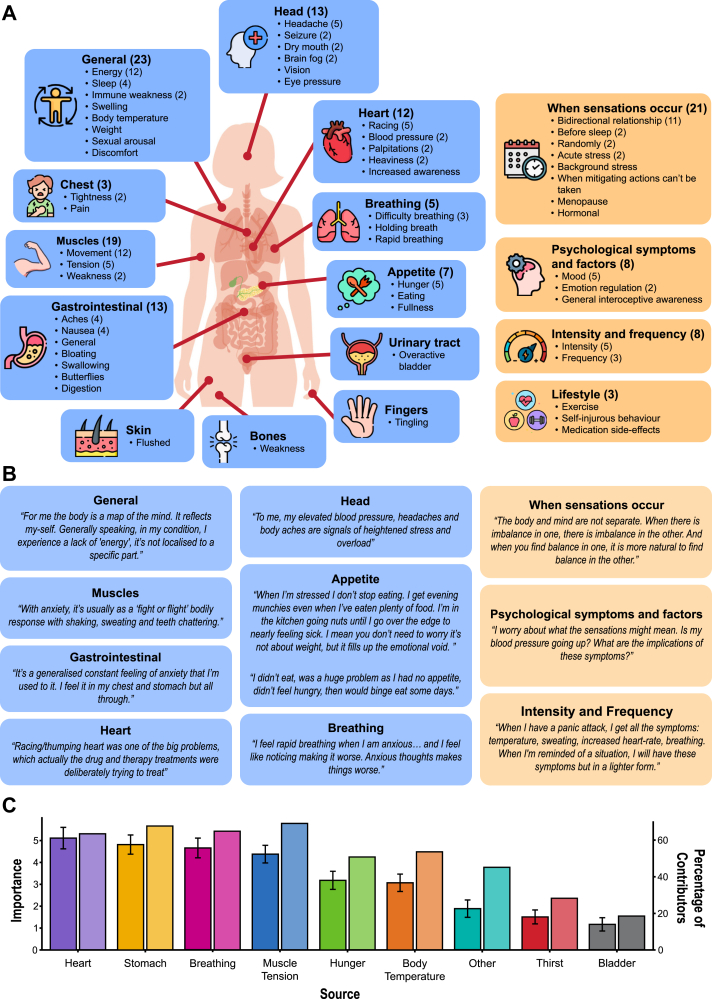
**The lived experience of the relationship between bodily sensations and mental health**. A. Themes arising in response to the question “How are body signals related to your mental health” (see Table S3 for details). This was asked prior to any input from researchers, meaning that not all responses fit into the fields' definition of interoception. Contributors could enter multiple answers, and answers could be categorised into multiple themes. Themes related to the location of bodily sensations are shown on the left in blue; other themes are shown on the right in yellow. The number in brackets refers to the frequency of each item being mentioned by contributors (if greater than one). B. Quotes displayed were drawn from the in-person workshop and were chosen to illustrate the emerging themes. C. Importance scores and prevalence of concern for different bodily sources, using combined data from both samples. For each source, the left-hand bar shows the mean of contributors' importance rankings, with standard error bars. Contributors ranked sources by ordering the ones they experienced from most to least important. The first source in their list was ranked highest and received a score of nine (there were nine total sources). The second score in their list was ranked second, receiving a score of eight, and so on. If a source was not selected by a contributor, it received a score of zero. Importance is the mean of the score a particular source received. A higher score indicates that, on average, the source was rated as more important. The right-hand bar shows the percentage of contributors who identified that source as a problem for them. The ‘Other’ category was free-text, and included: Blood pressure, brain fog, cognition, cold/flu symptoms, diabetes glucose levels, dizziness, dry mouth, fatigue, feeling faint, fidgeting, general internal reflection, headache, involuntary movements (shaking, teeth chattering, stuttering, tics), joint pain, non-epileptic seizures, pins and needles, skin, sweating, tears, throat tension, vision. It should be noted that not all of these free-text responses can be considered interoceptive, and thus caution should be used when interpreting the ‘other’ category.

Prior to any detailed information about the aims of the workshop (to keep responses independent of our and the fields’ perceived notions of interoception), in-person contributors were asked to provide insights into “how bodily signals are related to your mental health”. Qualitative analysis revealed five themes arising from these initial thoughts: (1) *location* of bodily sensations; (2) *when* sensations occur; (3) *psychological* symptoms and factors; (4) *intensity and frequency* of sensations, and (5) *lifestyle factors*. See Fig. 1A for a full breakdown of themes and sub-themes, with example quotes in Fig. 1B.

The vast majority of responses identified bodily sensations *localised* to a particular source: contributors reported a broad range of gastrointestinal symptoms (e.g., aches, nausea, bloating), compared to a narrower set of cardiac sensations which were typically specific to high arousal (e.g., racing heart). There were also numerous reports of less-localisable aspects of bodily sensations: sensations deriving from muscles and/or movement, such as fidgeting, tics, or clumsiness; or headaches, flushed skin, and ‘bone weakness’.

In identifying *when* these bodily sensations occur, people differed in how predictable they felt their experiences of sensations were. Several patients suggested that bodily symptom severity (i.e., *frequency* and *intensity*) increased in line with *psychological* symptoms and factors, including with anhedonia, low mood, and difficulty regulating emotions. Finally, patients identified specific *lifestyle factors*– behaviours that were associated with worsening experiences of bodily sensations, such as lack of exercise.

### Experiences of bodily sensations

#### Quantitative analyses

Contributors in both the workshop and online samples selected which bodily sources impacted them personally, and ranked these sources in order of importance. This list was adapted from Nord and Garfinkel^9^ and focused on interoceptive signals that are widely recognised in the field. Pain and fatigue were not explicitly listed due to their complex and potentially non-interoceptive origins, though both are referred to when describing sources in initial thoughts, such as muscles or the stomach. Contributors were also free to add additional signals, including these, into the ‘other’ option provided. The mean number of sources that were selected and ranked was 4.61 [1.93] (mean [standard deviation]), and no contributor selected no sources or all 10. Mean distress level (on a scale from one to ten) was 6.79 [2.24].

Fig. 1C depicts the percentage of contributors who selected each source and the mean rank of each source (or “importance score”). In particular, muscle tension, stomach, breathing and heartbeat were highly-selected and ranked by contributors. Interestingly, there are discrepancies between highly-selected and highly-ranked sources; for example, whilst muscle tension was selected by the highest percentage of contributors, heartbeat had the highest mean rank.

The vast majority of results were consistent across a range of subgroups (depression/no depression; GAD/no GAD; physical health condition/no physical health condition; men/women; neurodivergent/neurotypical; workshop/online); further information can be found in Supplementary Material 4.

#### Qualitative analyses

From the small group discussions, we identified four main themes: (1) *understanding aetiology*; (2) *attention to sensations*, (3) *factors that relate to sensations*, and (4) the *effects of sensations*.

##### Understanding aetiology

Discussions centred on a desire to understand the causes of bodily sensations—“[you're] told it's all in your head […] it would be nice sometimes to just find out something solid”—as well as frustration at the inability to predict them: “[it] makes me angry that it seems random”. An understanding of sensations was viewed as helpful for management: “when I can recognise the signs that are associated with mental health, that awareness helps me deal with it in advance”. Patients raised the difficulty of attributing the cause to a physical or mental origin, especially when physical and mental conditions interact: “asthma symptoms and breathing, when having an episode symptoms can be the same”.

##### Attention to sensations

Contributors reported a variety of impacts of paying attention to bodily signals. Some contributors found that directing attention to bodily signals can reduce distress, while others saw it as unrelated, saying that mindfulness-based bodily awareness alone “doesn't change the racing heart, doesn't calm [them] down”, or that it “doesn't change any of the actual quality of the illness”. Still others found that paying attention to bodily signals “only makes it worse”, that “looking into [their] body feels more anxious, it is almost like a self-inflicted wound”, and that distractions provide relief. As well as consciously altering their extent of body monitoring, some contributors reflected on their use of external monitoring devices (e.g., smart watches) to obtain objective measurements of bodily signals. Whilst some reported an increase in anxiety through obtaining this objective information, others noted benefits: “in the middle of a panic attack it can be reassuring to see that your heart isn't actually going that fast”. Overall, there was uncertainty over the optimal level of paying attention, with contributors struggling to “find the balance between healthy awareness of what's going on and being hypervigilant”.

##### Factors that relate to sensations

Environmental triggers included intense and stressful situations: “if you've been in an environment before that has triggered you, then going back into that environment again can push things up”, which led some people to have “avoided doing things [they] want to do because of managing triggers”. Hormones also played a role, both cyclical effects “that are connected to [the] menstruation cycle” and the effect of “fluctuations during perimenopause on mood and anxiety”. For some, sensations “varied from day to day”, while others experienced a steady worsening over time. The intensity of the environment can push back the effects of sensations on mental health, for one contributor “in an environment that is high stress and high pace […] the effect of being sick on [their] mental health is delayed. [They] have to put it on the back burner”, though they noted that this catches up to them, adding “once my mind runs out of things to occupy itself, I collapse a bit more”.

##### Effects of sensations

Some contributors interpreted potentially ambiguous sensations as signs of health problems, saying that “heart racing means I'm going to die”, or that they “automatically deem that any ache, pain, sickness or feeling of malaise is worse than it actually is”. This “vicious cycle” can be made worse when combined with the difficulties in understanding aetiology seen above. Symptoms associated with depression could trigger anxiety: “breathing slows, sometimes [I] make myself anxious because I'm worried about the slow rate of my heart”. While low levels of anxious energy was seen as potentially positive: “[it] helps me ride the wave”, there was a certain “tipping point” where things became distressing: “once it becomes panic, I have a line in my head and when it reaches that it goes haywire”. Other effects included the social stigma of visible sensations—“I find it really embarrassing and people notice [hot flushes]”—as well as difficulties falling asleep and unwanted behaviours such as “[staying] in bed for days on end”.

### Interactions with the healthcare system

We asked contributors from both samples: “When you have attended healthcare appointments relating to your mental health, has the relevance of bodily signals ever been discussed?” The majority had discussed bodily signals (Yes, bodily signals and ways to manage them: 35%; Yes, bodily signals: 26%), and a large number of those had found these conversations helpful (88% of the former, 53% of the latter). The workshop sample had the opportunity to elaborate on the topics discussed in these appointments. Those who found their experience helpful noted specific management techniques such as breathing exercises, grounding strategies, mindfulness, exercise and diet change. Others mentioned that it was helpful to understand how their mental and physical health impacted each other. One contributor discussed the reassurance they felt through reframing fatigue: “When I was tired all the time when I was depressed my psychologist recommended to just accept it and acknowledge that my body is doing a lot of emotional work and needs to rest from that. I found that really reassuring because it took away the fear that I'm being lazy”. Those who had not had these conversations pointed to the traditional view that mental and physical health are distinct and unrelated. As one contributor said, “I don't think doctors in the past recognised the links with mental health and I did not feel I could discuss this with them”. This highlights a common criticism that the healthcare system is siloed into different specialities and would benefit from taking a holistic approach.

Contributors were also asked: “In appointments for physical health issues, have you ever discussed the impact of physical health on your mental health?” Just over half of contributors reported having these conversations (53%), and only 61% of those found the conversations helpful. Most of the workshop elaborations on this question were from those who had had negative experiences. One contributor found it difficult that different specialists disagreed on whether physical or mental health conditions were their primary condition: “One neurologist suggested going down the route of diagnosing anxiety on the back of my physical conditions and making anxiety my primary diagnosis. The other neurologist suggested that I find a better gastroenterologist before I go down the route of attributing my physical symptoms to anxiety”. Another reported that discussions of whether symptoms were psychosomatic had been damaging: “my GP felt that my chronic pain was in my mind–of course this made me worse”. By contrast, a positive experience was one which highlighted the benefits of taking a wider view on the ways to manage the effects of their physical health: “A nutritionist (who I saw for IBS symptoms) recommended that I try therapy rather than just changing/controlling my diet”. This further highlights the importance of productive conversations and the need for improved integration between healthcare providers and specialties.

### Research priorities

We thematically analysed workshop contributors’ text-based responses on research priorities. These could be grouped into three broad themes: *causes* (origins of bodily sensations and their interactions); *management* (accurate identification and attention toward bodily sensations); and *approach* (clinical and research paradigms that could better advance research/treatment on this topic). The full list of priorities identified are shown in Supplementary Material 3 (Figure S1A).

We then analysed the online contributors’ ratings of the research priorities. All of the research priorities had mean ratings higher than six out of ten (Supplementary Material 3; Figure S1B). For all but one research priority, at least 50% of the contributors gave a rating of seven or above (Supplementary Material 3; Figure S1C). Ratings across a wide range of subgroups did not significantly differ (see Supplementary Material 4; note a gender difference for one research priority). Taken together, this suggests wide applicability of the research priorities identified.

Finally, we synthesised these research priorities into a list of *ten patient-led priorities for interoception research in psychiatry* (Supplementary Material 3):

Causes

1. How do mental health, physical health and bodily signals influence each other, and what are the gaps in this research?
2. How do distressing bodily signals impact peoples' ability to engage in social contexts?
3. How do bodily signals differ between individuals, and between different mental health conditions?

Management

4. What techniques can be used to manage distressing bodily signals?
5. How can people stop distressing bodily signals distracting them from daily life tasks?
6. How can people know if distressing bodily signals are a sign of an urgent physical problem (e.g., heart condition) compared to being a symptom of their mental health?
7. How can people more accurately identify their bodily signals?
8. What is the correct balance between paying attention to bodily signals and trying to distract from them?

Approach

9. Doctors and researchers should consider the body and mind together in their work.
10. People with lived experience of mental health conditions should be actively involved in the full process of research, from conceptualisation to dissemination.

## Discussion

It is now well-established that interoception plays a key role in mental health.^1^^,^^3^^,^^5^^,^^6^^,^^9^^,^^34^ Yet this work—including our own—has often neglected the priorities of people with lived experience, which may or may not accord with scientifically-driven research priorities. To remedy this, we used mixed methods to explore how bodily sensations are related to patients’ mental health, and collated patient generated-and-validated research priorities in the interoception field to support current work and highlight novel research areas.

Our findings emphasise the multimodal nature of the connection between the body and mental health: research should broaden beyond the current focus on cardiac and respiratory interoceptive modalities. This is particularly important given that interoceptive findings show poor correspondence across modalities,^12^ or even across tasks within a modality,^35^ meaning classic findings cannot necessarily be applied more generally. Patients highlighted a wide range of bodily sensations in *initial thoughts*, including muscle tension and the stomach, neither of which have received as substantial research attention as cardiac and respiratory modalities to date. Assessing gastric interoception has historically been challenging due to difficulty accessing the stomach. Electrogastrography, however, has been used in psychiatric populations, including in combination with neuroimaging to assess stomach-brain coupling.^36^ This result also reinforces the importance of novel techniques for gastric measurement and perturbation, including ingestible vibrating capsules^37^ and pharmacological targeting of gastric state.^38^ In contrast, muscle tension is not universally considered an interoceptive signal, and this categorisation may have compounded the experimental difficulties it poses. Extant muscle tension and muscular effort tasks focus on the ability to discriminate between various weights.^39^^,^^40^ However, patients’ experiences extend beyond weight discrimination. For example, contributors in our sample reported severe long-lasting muscle tension, something which could be explained by an increase in muscle tension itself or perceptual amplification due to heightened interoception; current work is unable to disambiguate these explanations. Future research on muscle tension should also investigate mechanisms of treatments which directly target this bodily signal (e.g., body scan; yoga), given the widespread positive reports of these approaches.

Our results suggest the focus of the field should be on a wide range of facets of interoception. Experimental work has typically measured interoceptive dimensions of accuracy, sensibility, and metacognition.^41^ Whilst insightful, these dimensions do not provide the full picture. Qualitative analyses of small group discussions indicated that people with mental health conditions are also concerned by the origins and interacting factors of bodily sensations, the role of paying attention to sensations, and the wider effects of bodily sensations on quality of life. These aspects move beyond the measures obtained in conventional experimental assessments and into a context-centred perspective; this approach would be facilitated by the use of ecologically valid measures such as experience-sampling^42^^,^^43^ and behavioural assessments that can be completed outside of laboratory settings.^44^^,^^45^ The aim to broaden the study of interoception accords with similar research priorities in the field: Suksasilp and Garfinkel^46^ include attribution as an important dimension of interoception, while Khalsa and colleagues^1^ highlight ways to measure the perceived intensity of interoceptive signals. In experimental work, MacCormack and colleagues^47^ found valenced beliefs about interoceptive signals moderated the link between physiological stress and emotional arousal. Finally, Murphy has reflected on the need to establish optimal levels of interoceptive attention,^48^ a priority raised by our contributors. Interestingly, studies of anxiety and interoceptive training have reported distinctions between the Body Perception Questionnaire (whose questions primarily focus on attention to negative internal sensations) and the Multidimensional Assessment of Interoceptive Awareness (which includes questions relating to positive attention to internal signals).^7^^,^^49^ This suggests that research addressing optimal levels of interoceptive attention should separately consider the ability of attention-based interventions (e.g., mindfulness) to *increase* positive processing and *decrease* negative processing. Overall, experimental research investigating the understudied aspects of interoception raised by our contributors may more accurately depict the true impact of bodily signals on patients’ health, and therefore provide an important link in understanding and treating mental health conditions.

The key contribution of this paper is the list of interoception-based research priorities generated and validated by people with mental health conditions. These research priorities focused on understanding of the causes of bodily sensations and identifying avenues for managing distressing sensations. More broadly, as well as the importance of communicating aims and incorporating lived experience expertise, patients highlighted the desire for a more holistic approach to research and clinical practice. Indeed, research creates boundaries between different theoretical concepts which are not necessarily reflected in an individual's experience (e.g., interoception versus proprioception), whilst clinical practice often fails to consider mental and physical health conditions together despite their common co-occurrence and interaction with one another.^50^ Efforts to establish cross-cutting research and clinical practice—something the interoception field is well-positioned to do—will improve treatment prospects and experiences of patients within the healthcare system. Overall, we recommend that researchers and funders direct attention to the research priorities identified whilst maintaining a cross-cutting approach.

We note several limitations. The foremost, applicable to all PPI work, is that it is suited to conscious priorities: contributors have to be aware of a problem to report on it. Therefore, it cannot directly address unconscious processing or metacognition, two important areas within interoceptive research. Our findings must be integrated into the wider literature in determining how best to understand and treat interoceptive issues in mental health. In addition, it is difficult to ensure that PPI is fully generalisable. For example, the large representation of women in our sample, whilst in line with population-level data reporting that women face a higher mental health burden than men,^51^ makes our findings less applicable to men. We made intentional efforts to recruit contributors who reflected the UK population in terms of ethnicity and mental health diagnoses, and to include a wide range of ages, physical health conditions and neurodiversities. Nevertheless, the limited size of this project, whilst necessary for the in-depth small-group workshops, precludes effective comparison between diagnoses or other subgroups (though note preliminary results in Supplementary Materials demonstrating widespread consistency on quantitative analyses for a range of subgroups). Our study took a transdiagnostic approach, representing a broad range of experiences and priorities in the psychiatry interoception field. Whilst this acts as a positive first step in a field devoid of such work, our approach does have its limitations. For example, the inclusion of people with multiple mental health diagnoses—something which is representative of real-world experiences^29^—means experiences cannot be attributed to specific mental health conditions. As such, we propose that our broadly generated research priorities should be used as a guide for research and that research projects should be supplemented by smaller-scale PPI activities to refine the research question for researchers’ specific fields. Finally, all contributors are adults, resident in the UK (with in-person contributors being local to the South of England), fluent in English, and socio-economic data were not recorded. This impacts the translation of findings to other contexts (e.g., children/adolescents and individuals residing in other countries).

In summary, this article presents novel insights into patients’ experiences of interoception in mental health, as well as specific priorities for future research. The results of this paper will help ensure that interoception research is informed by lived experience and aligned with the needs of those whose lives we are trying to improve. We hope that researchers and clinicians in interoception and psychiatry more broadly find useful ideas in this article, including novel research topics highlighted in the qualitative work, and research priorities that matter to people with mental health conditions. Ultimately, this work calls for a broad, comprehensive and holistic approach to researching the distressing experiences of bodily sensations in mental health conditions.

## Contributors

Conceptualization (L.J.H., G.M., S.N.G., C.L.N.); Methodology (L.J.H., G.M., B.L., H.S.S., S.N.G., C.L.N.); Investigation (L.J.H., G.M., B.L., H.S.S., E.B., H.F., R.K., I.L., A.W., S.N.G., C.L.N.); Data curation (L.J.H., G.M., E.B., H.F., R.K., I.L., A.W.); Formal analysis (L.J.H., G.M., C.L.N); Funding acquisition (S.N.G., C.L.N.); Project administration (L.J.H., G.M.); Software (L.J.H., G.M.); Supervision (S.N.G., C.L.N.); Visualization (G.M.); Writing–original draft (L.J.H., G.M., C.L.N.); Writing–review & editing (L.J.H., G.M., B.L., H.S.S., E.B., H.F., R.K., I.L., A.W., S.N.G., C.L.N.). All authors read the final manuscript and approved the submission. All authors had access to the data. L.J.H. and G.M. verified the data.

## Data sharing statement

The quantitative data and analysis scripts are available on OSF (https://osf.io/ygwcq/?view_only=b1f63128c1fa4200913848671ca7aa3c).

## Declaration of interests

The authors declare no conflict of interests.
