# Supplementary material for: Breaking through the mind-body divide: patient priorities for interoception research
Source: eClinicalMedicine. 2025 Mar 31;82:103183. doi: 10.1016/j.eclinm.2025.103183 (PMC11999071; doi:10.1016/j.eclinm.2025.103183)
Supplement: Supplementary Materials [file mmc1.docx]

**SUPPLEMENTARY MATERIAL FOR**

**“Breaking through the mind-body divide: patient priorities for interoception research”**

**Table of contents**

[**Supplementary Material 1. Additional demographic information** 2](#_Toc191995477)

[**Supplementary Material 2. Study questions.** 4](#_Toc191995478)

[**Supplementary Material 3. Research priority generation, validation and finalisation.** 6](#_Toc191995479)

[**Supplementary Material 4. Subgroup analyses** 8](#_Toc191995480)

## **Supplementary Material 1. Additional demographic information**

**Table S1. Physical health conditions diagnosed in the combined, workshop and online samples**

| **Characteristics** |  | **Combined** | | **Workshop** | | **Online** | |
| --- | --- | --- | --- | --- | --- | --- | --- |
| **Physical Health** |  |  |  |  |  |  |  |
|  | **None** | 30 | (42%) | 11 | (44%) | 19 | (40%) |
|  | **Gastrointestinal Disorders** | 12 | (17%) | 5 | (20%) | 7 | (15%) |
|  | **Pain Disorders** | 12 | (13%) | 5 | (16%) | 7 | (11%) |
|  | **Cardiovascular Conditions** | 10 | (14%) | 4 | (16%) | 6 | (13%) |
|  | **Musculoskeletal Disorders** | 7 | (10%) | 2 | (8%) | 5 | (11%) |
|  | **Neurological and Psychological Conditions** | 7 | (7%) | 3 | (8%) | 4 | (6%) |
|  | **Endocrine and Metabolic Disorders** | 6 | (8%) | 2 | (8%) | 4 | (9%) |
|  | **Respiratory Conditions** | 4 | (6%) | 2 | (8%) | 2 | (4%) |
|  | **Blood Disorders** | 3 | (3%) | 0 | (0%) | 3 | (4%) |
|  | **Autoimmune and Inflammatory Conditions** | 2 | (3%) | 0 | (0%) | 2 | (4%) |
|  | **Other** | 2 | (3%) | 1 | (4%) | 1 | (2%) |
|  | **Prefer not to say** | 2 | (3%) | 0 | (0%) | 2 | (4%) |
|  | **Sensory and Developmental Conditions** | 2 | (3%) | 1 | (4%) | 1 | (2%) |
| **Number of Diagnoses** |  |  |  |  |  |  |  |
|  | **0** | 30 | (42%) | 11 | (44%) | 19 | (40%) |
|  | **1** | 26 | (36%) | 9 | (36%) | 17 | (36%) |
|  | **2** | 6 | (8%) | 1 | (4%) | 5 | (11%) |
|  | **3** | 5 | (7%) | 3 | (12%) | 2 | (4%) |
|  | **4** | 1 | (1%) | 0 | (0%) | 1 | (2%) |
|  | **5** | 2 | (3%) | 1 | (4%) | 1 | (2%) |
|  | **Prefer not to say** | 2 | (3%) | 0 | (0%) | 2 | (4%) |

*Note*. For physical health: count indicates the number of diagnoses in the sample that fall under the category; number in brackets represents the percentage of contributors in the sample with at least one diagnosis that falls under the category. For number of diagnoses: count indicates how many contributors in the sample have the specified number of diagnoses; number in brackets is the count as a percentage. Each category contains the following conditions - Gastrointestinal Disorders: Irritable Bowel Syndrome, Barrett's Syndrome, Hiatus Hernia; Pain Disorders: Chronic Pain, Fibromyalgia; Cardiovascular Conditions: Hypertension, Borderline Hypertension, Heart Condition; Musculoskeletal Disorders: Osteoporosis, Osteoarthritis, Osteopenia, Arthritis, Rheumatoid Arthritis; Neurological and Psychological Conditions: Chronic fatigue, Migraines, Neurological Migraines, Dissociative seizures, Functional Neurological Disorder; Endocrine and Metabolic Disorders: Polycystic Ovary Syndrome, Diabetes, Hypothyroidism, Obesity; Respiratory Conditions: Asthma, Bronchiectasis; Blood Disorders: Pernicious Anemia, Thrombocytopenia, Thrombophilia; Autoimmune and Inflammatory Conditions: Sarcoidosis, Endometriosis; Sensory and Developmental Conditions: Mild hearing difficulty, Microphthalmia; Other: Cancer Remission, Physical Impairment

**Table S2. Neurodiversity of the combined, workshop and online samples**

| **Characteristics** |  | **Combined** | | **Workshop** | | **Online** | |
| --- | --- | --- | --- | --- | --- | --- | --- |
| **Neurodivergence** |  |  |  |  |  |  |  |
|  | **None** | 46 | (64%) | 20 | (80%) | 26 | (55%) |
|  | **ADHD** | 12 | (17%) | 3 | (12%) | 9 | (19%) |
|  | **Autism** | 5 | (7%) | 0 | (0%) | 5 | (11%) |
|  | **Prefer not to say** | 6 | (8%) | 1 | (4%) | 5 | (11%) |
|  | **Dyslexia** | 2 | (3%) | 0 | (0%) | 2 | (4%) |
|  | **Other** | 2 | (3%) | 0 | (0%) | 2 | (4%) |
|  | **Dyspraxia** | 1 | (1%) | 1 | (4%) | 0 | (0%) |
| **Number of Diagnoses** |  |  |  |  |  |  |  |
|  | **0** | 46 | (64%) | 20 | (80%) | 26 | (55%) |
|  | **1** | 18 | (25%) | 4 | (16%) | 14 | (30%) |
|  | **2** | 2 | (3%) | 1 | (4%) | 2 | (4%) |
|  | **Prefer not to say** | 6 | (8%) |  |  | 5 | (11%) |

*Note*. For neurodivergence: count indicates the number of reports of the condition in the sample; number in brackets represents the percentage of contributors in the sample with the condition. For number of diagnoses: count indicates how many contributors in the sample have the specified number of diagnoses; number in brackets is the count as a percentage. Eight conditions reported in the Combined sample were not officially diagnosed. The ‘Other’ category contains the following conditions: Highly Sensitive Person (HSP), Unsure/Undiagnosed. ADHD = Attention Deficit Hyperactivity Disorder.

## **Supplementary Material 2. Study questions.**

**Table S3. Questions asked in the workshop and online samples**

| Section | Workshop questions | Online questions |
| --- | --- | --- |
| Initial thoughts | Presentation prompt: How do you think the body is involved in mental health? What bodily signals have you noticed? Where are they in the body? When do you experience them?  Slido question: How are body signals related to your mental health? |  |
| Signals discussion | Presentation prompt: How are bodily signals affected? Strength: too strong / too weak. Attention: too much / not enough. Meaning: worried / can’t work it out. Where are they in the body? Chest (heart, lungs); limbs; stomach; head. When do you experience them? Trying to sleep; Stressful situations; Exercising; Working; Specific points of the menstrual cycle; Resting; All the time |  |
| Signals and treatment – quantitative questions | What signals are issues for you? Select all that apply.   - Heartbeat; Breathing; Stomach; Bladder; Hunger; Thirst; Body Temperature; Muscle Tension; None; Other (please specify)   Rank how important you think each of these signals is to research. You can drag the signals to order them.   - Heartbeat; Breathing; Stomach; Bladder; Hunger; Thirst; Body Temperature; Muscle Tension; None; Other (please specify)   How distressing are bodily signals in relation to your mental health conditions (where 1 = not distressing and 10 = highly distressing)?  When you have attended healthcare appointments relating to your mental health, has the relevance of bodily signals ever been discussed?   - Yes – bodily signals; Yes – bodily signals AND ways to manage them; No; I’m not sure   Was the information helpful?   - Yes; No; Not Applicable   WORKSHOP ONLY: What topics did you discuss? (if you would like to share)  In appointments for physical health issues, have you ever discussed the impact of physical health on your mental health?   - Yes; No; I’m not sure   Was the information helpful?   - Yes; No; Not Applicable   WORKSHOP ONLY: What topics did you discuss? (if you would like to share) | |
| Research priority generation | What question would you most want answered about the involvement of body signals in mental health conditions? |  |
| Research priority validation |  | Below are a list of research questions we could focus on. Please indicate how important you think each possible research question is based on your own experiences of mental health (0-10).  Understanding the causes of issues   - How do mental health, physical health and bodily signals influence each other? - How do distressing bodily signals impact peoples’ ability to engage in social contexts? - Why does psychological distress cause physical symptoms? - How do bodily signals differ between individuals, and between different mental health conditions?   Dealing with issues   - What is the correct balance between paying attention to bodily signals and trying to distract from them? - How can people more accurately identify their bodily signals? - Can medication help people cope with distressing bodily signals? - How can people stop distressing bodily signals distracting them from daily life tasks? - Why do people do harmful things to cope with distressing bodily signals? - What new techniques can be developed for managing distressing bodily signals? - How can people know if distressing bodily signals are a sign of an urgent physical problem (e.g., heart condition) compared to being a symptom of their mental health? - Where can people learn about existing techniques for managing distressing bodily signals?   How we should do research   - Researchers should communicate the purpose of their studies to people with lived experience of the conditions they are studying - Doctors should find ways to better consider the body and mind together when treating people - Researchers should ensure that they better consider both the body and mind together when researching mental health - People with lived experience of mental health conditions should be more involved in setting the aims of research - Gaps in research of bodily signals and their relationship to mental health should be identified |

## **Supplementary Material 3. Research priority generation, validation and finalisation.**

The full list of research priorities generated by participants can be seen in Figure S1A. These research priorities were validated by the online sample who rated them between zero and ten (where zero = “not at all important” and ten = “extremely important”). Figure S1B presents the mean ratings for each research priority, whilst Figure S1C depicts the proportion of contributors rating each research priority as below four, four to six, and over six.

**
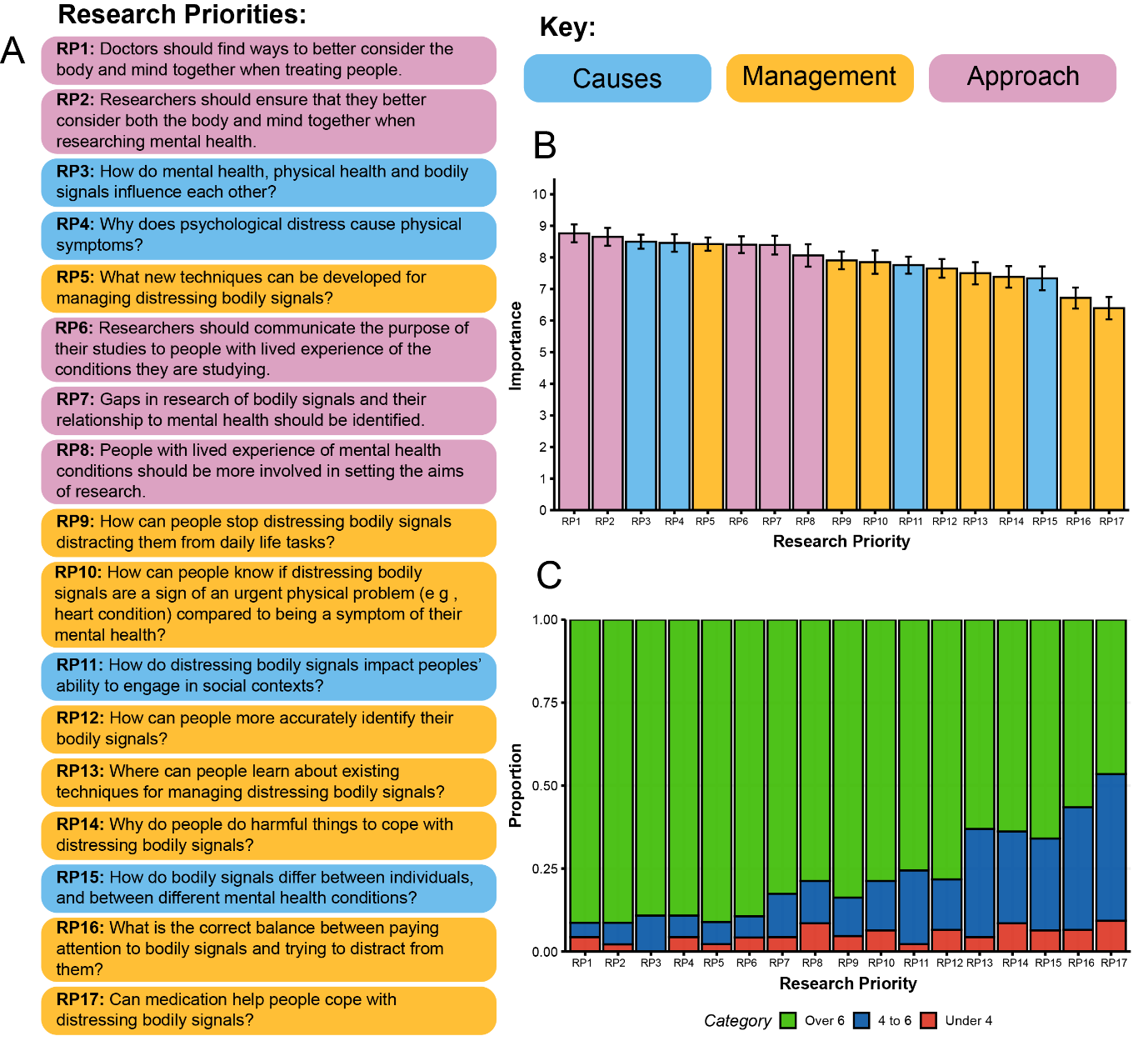
Figure S1. Research priorities for interoceptive research generated and validated by individuals with lived experience.**

*Note.* A) The research priorities generated following a thematic analysis of contribution from the workshop sample, B) mean scores for each research priority in the validation phase completed by the online sample and C) levels of agreement among online contributors in the ranking of research priorities.

***Creating the final list of ten patient-led priorities for interoception research in psychiatry***

The full list of research priorities identified from contributors’ suggestions were synthesised into *ten patient-led priorities for interoception research in psychiatry*. These are presented below along with the original research priorities that were combined to create the final list of ten.

*Causes*

How do mental health, physical health and bodily signals influence each other, and what are the gaps in this research?

- *RP3 How do mental health, physical health and bodily signals influence each other?*
- *RP4 Why does psychological distress cause physical symptoms?*
- *RP7 Gaps in research of bodily signals and their relationship to mental health should be identified*

How do distressing bodily signals impact peoples’ ability to engage in social contexts?

- *RP11 How do distressing bodily signals impact peoples’ ability to engage in social contexts?*

How do bodily signals differ between individuals, and between different mental health conditions?

- *RP15 How do bodily signals differ between individuals, and between different mental health conditions?*

*Management*

What techniques can be used to manage distressing signals?

- *RP5 What new techniques can be developed for managing distressing bodily signals?
  RP13 Where can people learn about existing techniques for managing distressing bodily signals?*
- *RP14 Why do people do harmful things to cope with distressing bodily signals?*
- *RP17 Can medication help people cope with distressing bodily signals?*

How can people stop distressing bodily signals distracting them from daily life tasks?

- *RP9 How can people stop distressing bodily signals distracting them from daily life tasks?*

How can people know if distressing bodily signals are a sign of an urgent physical problem (e.g., heart condition) compared to being a symptom of their mental health?

- *RP10 How can people know if distressing bodily signals are a sign of an urgent physical problem (e.g., heart condition) compared to being a symptom of their mental health?*

How can people more accurately identify their bodily signals?

- *RP12 How can people more accurately identify their bodily signals?*

What is the correct balance between paying attention to bodily signals and trying to distract from them?

- *RP16 What is the correct balance between paying attention to bodily signals and trying to distract from them?*

*Approach*

Doctors and researchers should consider the body and mind together in their work.

- *RP1 Doctors should find ways to better consider the body and mind together when treating people*
- *RP2 Researchers should ensure that they better consider both the body and mind together when researching mental health*

People with lived experience of mental health conditions should be actively involved in the full process of research, from conceptualisation to dissemination.

- *RP6 Researchers should communicate the purpose of their studies to people with lived experience of the conditions they are studying*
- *RP8 People with lived experience of mental health conditions should be more involved in setting the aims of research*

## **Supplementary Material 4. Subgroup analyses**

Following Reviewers’ comments, we conducted subgroup analyses for the primary quantitative components in main text. Specifically, we report subgroup summary statistics for *experiences of bodily signals* and *research priority validation*. Table S4 reports the sample sizes for these two analysis subsections, as well as the mean number of sources selected and mean distress ratings for each subgroup. The sources’ importance scores and percentage of contributors selecting each source are presented in Figure S2, whilst research priority ratings are presented in Figure S3. In each case, the bar for each subgroup denotes the difference between the subgroup value and the overall value for that particular source or priority.

We created subgroups of presence or absence of the most common mental health diagnoses (depression: absent / present; GAD: absent / present), physical health condition diagnoses (present / absent), gender (men / women), neurodiversity (neurodivergent / neurotypical) and sample (workshop / online). It was not possible to conduct selective analyses for more specific mental health conditions due to the fact that 75% of the sample had more than one mental health diagnosis. Given the small sample size of these subgroups, interpretation of the below analyses warrants caution.

The vast majority of results were consistent between subgroup pairs. Post-hoc tests indicated no significant differences between subgroup pairs for mean number of sources selected and mean distress rating (Table S4). For importance ratings and the percentage who selected each source, the difference between subgroup pairs was non-significant for the vast majority of sources – assessed using non-parametric Brunner-Munzel tests, bonferrroni-corrected across sources (N = 9). Men had a lower mean importance score and selection percentage for *stomach* compared to women (with an importance score of 2.41 and 5.63 and percentage of 35% and 78% for men and women respectively, both comparisons p_bf_ < .05); given the correspondence between the two measures, this can be considered the most meaningful subgroup source difference. Further to this, a higher percentage of workshop contributors selected *other* compared to the online sample (75% and 30% respectively, p_bf_ < .05), though no significant difference in importance ratings was observed. We suggest that this difference may be due to factors specific to the workshop sample (e.g., prior in-depth discussions; a longer length of time to answer the question). Finally, *thirst* had a lower importance score in the depression-absent subgroup compared to the depression-present subgroup (0.19 and 1.89 respectively, p_bf_ < .05); given the low number of people in either subgroup who rated thirst as important to any degree, and the lack of correspondence in the percentage comparison, this result in particular should be treated with caution.

Subgroup differences for research priority validation were assessed using non-parametric Brunner-Munzel tests, bonferrroni-corrected across research priorities (17). No differences were found between subgroup pairs except in one instance: men rated research priority 2 (“Researchers should ensure that they better consider both the body and mind together when researching mental health”) lower than women (p_bf_ < .05), with ratings of 6.38 and 8.06 respectively.

**Table S4. Subgroup analyses sample information**

| **Group** | **Experiences N** | **RP validation N** | **Mean number of sources selected** | **Mean distress rating** |
| --- | --- | --- | --- | --- |
| **Overall** | 71 | 47 | 4.61 (1.93) | 6.79 (2.24) |
| **No Depression** | 16 | 9 | 4.12 (1.93) | 6.75 (2.29) |
| **Depression** | 55 | 38 | 4.75 (1.93) | 6.80 (2.24) |
| **GAD** | 29 | 19 | 4.66 (1.72) | 7.21 (1.76) |
| **No GAD** | 42 | 28 | 4.57 (2.09) | 6.50 (2.49) |
| **No Physical Condition** | 30 | 19 | 4.23 (1.79) | 6.50 (2.26) |
| **Physical Condition** | 39 | 28 | 4.87 (2.07) | 7.10 (2.23) |
| **Men** | 17 | 8 | 4.00 (1.54) | 6.18 (2.53) |
| **Women** | 51 | 37 | 4.75 (1.96) | 6.90 (2.12) |
| **Neurodivergent** | 20 | 16 | 4.95 (1.85) | 7.10 (1.48) |
| **Neurotypical** | 45 | 26 | 4.51 (1.95) | 6.64 (2.41) |
| **Workshop Sample** | 24 | 0 | 4.92 (1.74) | 7.00 (2.17) |
| **Online Sample** | 47 | 47 | 4.45 (2.02) | 6.68 (2.29) |

*Note*. For each group listed in the table, Experiences N denotes its sample size for the *experiences of signals* quantitative analyses, whilst RP validation N denotes its sample size for *research priority validation*. Sample sizes for subgroup pairs do not equal the overall sample sizes due to the following cases: Physical - “Prefer not to say” N = 2; Neurodivergent - “Prefer not to say” N = 6; Gender - “non-binary” N = 3. Mean number of sources selected and mean distress rating are reported as: mean (standard deviation). Minimum and maximum values are 0-9 and 1-10 respectively. For both variables, paired subgroups (e.g., depression-absent versus depression-present) were not significantly different as evaluated using non-parametric Brunner-Munzel tests (all p>.05). GAD = Generalised Anxiety Disorder, RP = Research Priority.

**Figure S2. Subgroup analysis of importance scores and prevalence of concern for different bodily sources.**


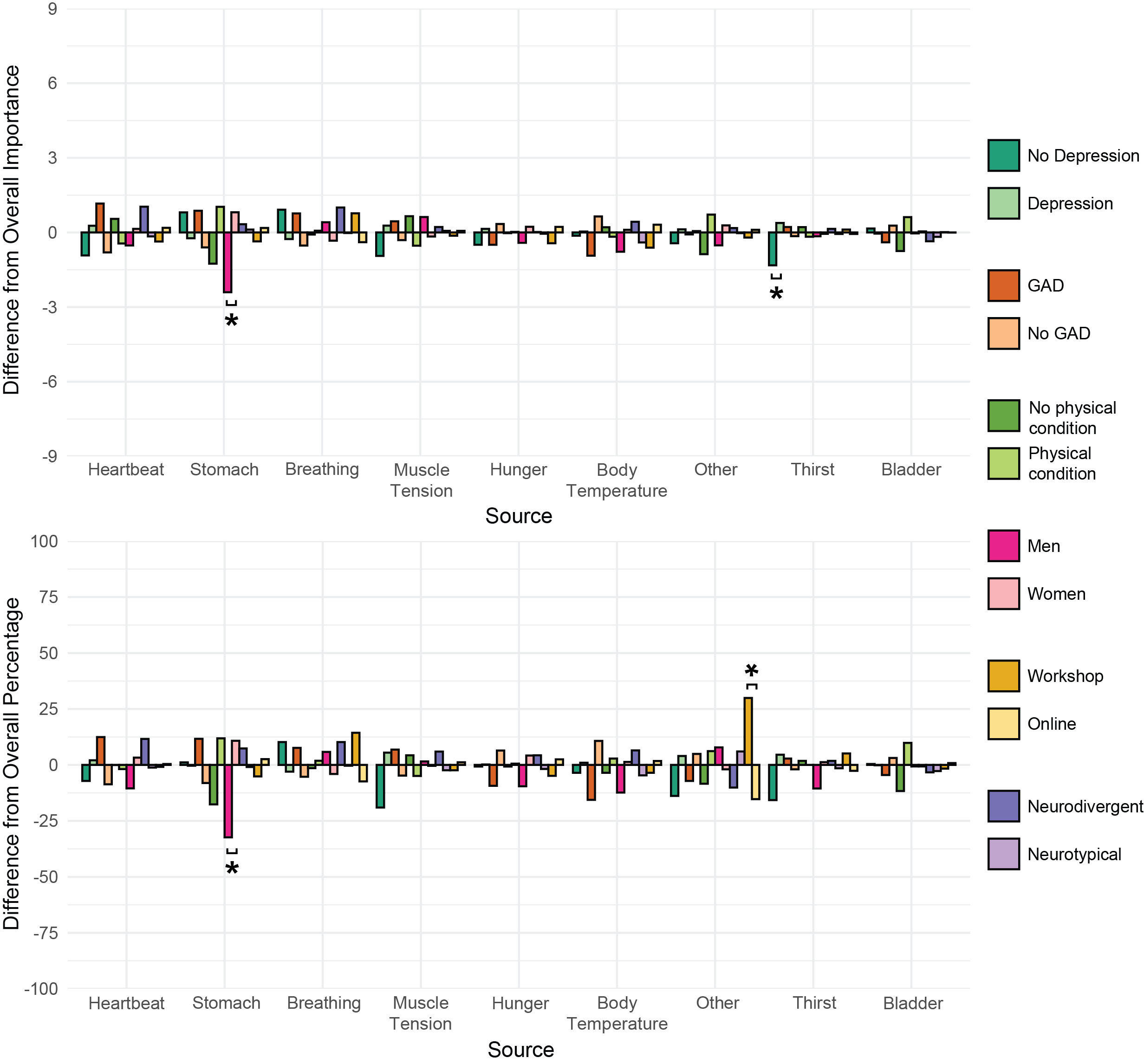


*Note*. Figure S2 (*top*) plots data relating to mean importance scores (calculated as the mean ranked position for selected sources; possible scores for each source range from zero to nine). Figure S2 (*bottom*) plots data relating to the percentage of contributors who identified that source as a problem for them (possible percentages range from 0% to 100%). In each case, the bar denotes the difference between the subgroup value and the overall value for the given source. The y-axes extend between the maximum and minimum differences scores that could be obtained. Fisher’s exact tests (for percentage selected) and non-parametric Brunner-Munzel tests (for importance scores) were run between subgroup pairs for each source (bonferroni-corrected for nine sources). Significant differences are indicated on the plot in which a ‘*’ denotes p<.05. Four differences were significant: men had a lower selection percentage and mean rank for *stomach* compared to women; a higher percentage of workshop contributors selected *other* compared to the online sample; *thirst* had a lower importance score in the depression-absent subgroup compared to the depression-present subgroup.

**Figure S3. Subgroup analysis of research priority validation.**


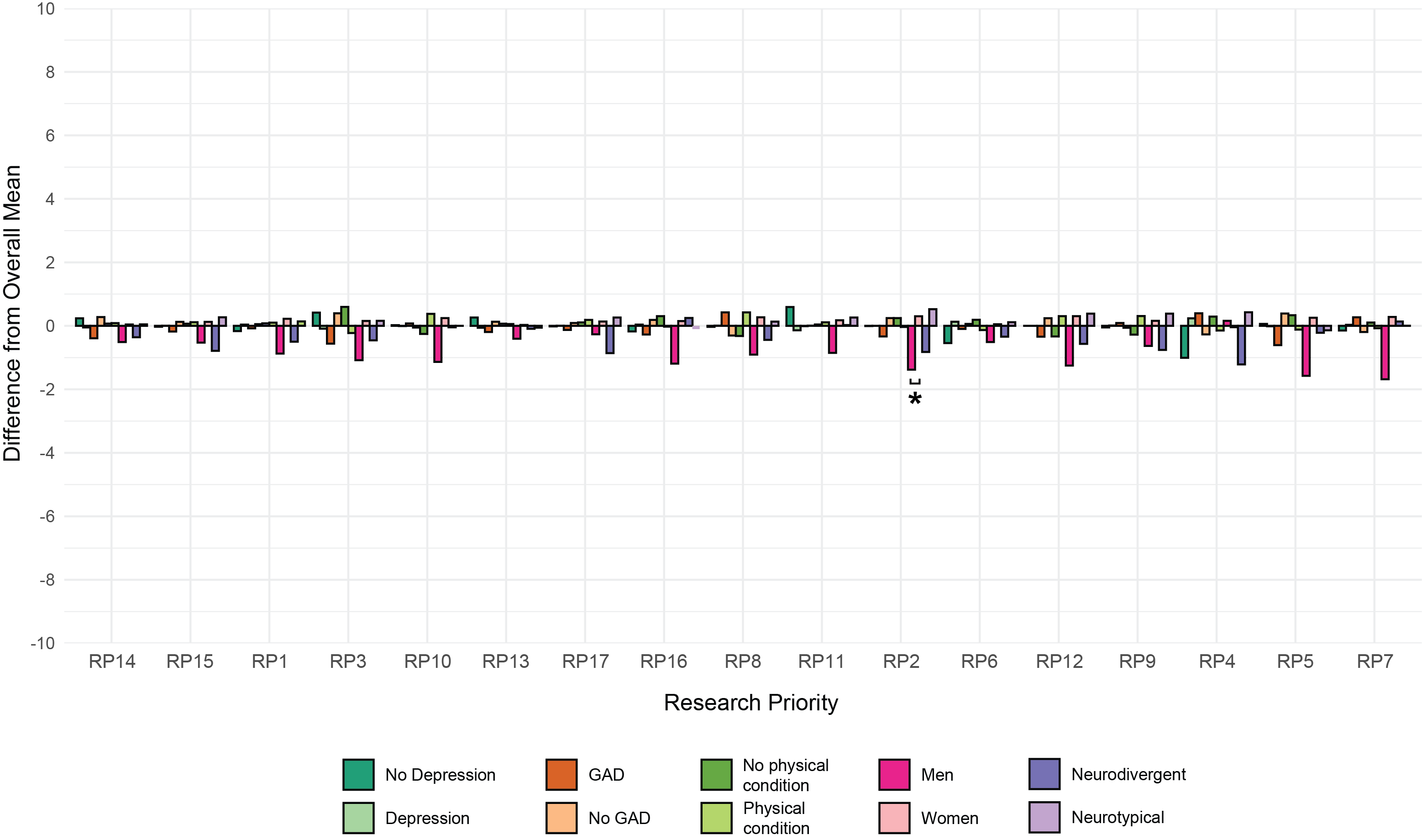


*Note*. The graph plots data relating to the ratings of each research priority (on a scale of zero to ten). Each bar denotes the difference between the subgroup mean rating and the overall mean rating for the given research priority. The y-axis extends between the maximum and minimum differences score that could be obtained. Non-parametric Brunner-Munzel tests were run between subgroup pairs for each research priority (bonferroni-corrected for 17 research priorities). Significant differences are indicated on the plot in which a ‘*’ denotes p<.05. There was one significant difference: men rated research priority 2 (“Researchers should ensure that they better consider both the body and mind together when researching mental health”) lower than women.
